# Observations on Palpitations of the Heart and Its Treatment

**Published:** 1871-06

**Authors:** Frederick B. Nunnely

**Affiliations:** London, Assistant Physician to the Hospital for Diseases of the Chest, Victoria-Park, and to the Hospital for Sick Children


*Observations on Palpitations of the Heart and its Treatment.* By
Frederick B. Nunnely, M.D., London, Assistant Physician to the Hospital for Diseases of the Chest, Victoria-Park, and to the Hospital for Sick Children.

---

The frequency of the disease, and the disproportion often existing between the sensation complained of and the physical signs of disordered action of the heart, have led to the following remarks, in which the term palpitation refers only to the patient's sensation, and is thus used with its popular, though incorrect, signification.

Palpitation is one of those symptoms of a deviation from health, regarded by the subject of it with anxiety, or even with alarm, but of the nature and seat of the cause of which we only possess a slight knowledge, in so far as this consists in a structural alteration of some part of the nervous system,

A further difficulty arises from the fact that palpitation, disorderly or perverted action of the heart, and structural changes of the organ, are not always quite clearly distinguished from one another, and that a definite relationship between structural change or disorderly movement on the one hand, and palpitation on the other, is *necessarily* supposed to exist, whilst there is often no proportion between the inconvenience and pain which the patient suffers, and the signs of perverted action which can be detected by physical examination. The patient has the unnatual and uneasy cognizance of the action of his heart, and from this he seeks release, and is not unfrequently grateful for the cure of a disease of which he only knew the existence; in other words, a "beating of the heart" may be complained of when no deviation from its natural action can be detected; or there may be grave structural disease, and the most disorderly action, and yet the patient may make little or no complaint.

It is quite true that in many cases the heart *throbs* in a manner evident to the observer as well as the patient; but in a considerable number the patient only can assert such to be the case. Palpitation is so often a mere symptom which, like pain, though quite real, varies in intensity with the individual, that it may be practically regarded as the *subjective appreciation of the heart's action.* Just as a patient who suffers pain after food—"knows that he has a stomach"—is the subject of dyspepsia, so a patient who feels the beating of his heart—"knows that he has a heart"—suffers from palpitation.

In health the heart beats with a frequency varying within narrow limits with each individual, the contractions are performed with regularity, and in definite order, best expressed by the term rhythmical. Any deviation from this state is a *derangement* of the heart's action, and affects either the frequency, order, duration, or intensity of its movements, and the knowledge of such alteration on the part of the subject of it, or even of the normal action, constitutes *palpitation*. Changes in point of frequency and of intensity, whether real or apparent, are more usually associated with sensation than those of order and duration, so that in a case of palpitation, if any physical signs exist, there will probably be increased frequency and force of the heart's contractions.

The pathology of palpitation, as of perverted movement, is at present little known. There is no doubt, however, that it is to be studied in the altered structure of the nervous ganglia and plexuses connected with the heart, the centres of the vagi and sympathetic nerves, and the base of the brain and spinal cord.

The exciting causes of palpitation are the various attitudes of mental and bodily excitement; over-work of the heart, as in severe physical labor; mechanical displacement of the organ; certain conditions of the blood, whether naturally acquired or due to the introduction of substances from without; inflammation of the heart; reflex causes, as impressions on other organs having a nervous connexion with the heart; and also certain conditions which, like those giving rise to attacks of neuralgic tic, are for the most part at present unknown.

A scientific classification of cases of palpitation is not at present possible from our slender knowledge of its pathology, and from the want of proportion between the disease and its physical signs. The following arrangement is simply one of convenience, as it throws together cases more or less resembling one another in their clinical history and progress.

(*a*) Cases occurring in persons free from structural disease of the heart, and of any organ having a nervous connexion with it. Two classes of these patients are met with—the young in whom degenerative changes have not commenced, and those in middle or advanced life in whom they have made considerable progress.

1. Palpitation occurs in young persons of both sexes, but especially in women, in whom it is often associated with pleurodynia. A mild form of irritative dyspepsia, and paleness not amounting to anæmia, are sometimes present. There is a history of impaired health, often dating from the time of the commencement of the catamenia, or of some moral shock in the nature of an affliction or disappointment, and a "beating at the heart" is complained of on the least exertion or excitement. The touch detects increased force of the heart's impulse; to the ear there is augmented intensity or abruptness of the sounds, and their frequency often exceeds that of health. Usually there is no irregularity, but now and then there is an intermission, or one or other sound is reduplicated. In these cases there would appear to be exalted susceptibility of the heart to emotional impressions, or to trifling demands of extra work, such as is required in going up stairs, together with impaired nutrition of its nerves.

2. Palpitation occurring in middle or advanced life, apart from other diseases, is probably one expression of that degeneration affecting the nervous system which is at this period of life making progressive inroads on the integrity of every structure of the body. The clinical history of such cases accords with this view. The palpitation is often of long standing, and has come on gradually, appearing after some serious illness, recovery from which has been slow, or is ascribed to some heavy affliction which has impaired the health. It is more common in women than in men, and sometimes dates from the cessation of the catamenia. The least exertion or excitement brings it on; it often disturbs sleep, is essentially chronic, and is apt to recur after apparent cure. Physical examination reveals a weak diffused impulse, irregular in force and frequency, and often with intermissions; or it may be that the action is regular, but somewhat abrupt. Usually, however, the regular, abrupt impulse of earlier life is absent. These patients are often the subjects of dyspepsia, due probably to a degeneration of some of the glandular structures of the stomach, but the palpitation will persist long after the digestion has been fairly restored by the adaptation of diet and exercise to the lessened power of the stomach.

(*b*) Cases of *distinctly* reflex origin. These are few in number, and are chiefly the results of the ingestion of food improper in quantity and quality, removal of which gives speeds relief. Probably, as our knowledge increases, this class will be largely augmented.

(*c*) Palpitation, associated with structural disease of the heart or its valves, occurs at all ages. It is not a prominent symptom of these diseases (except of fatty generation), in the ordinary use of the term, which includes changes of the valves, causing murmurs, obvious hypertrophy, or dilatation sufficient to lead to the grave consequences of over-filling of the venous system, and appears to be rather the effect of impaired nutrition of the heart and its nerves than of the mechanical faults of the organ. Its characters are those of the palpitation of middle life previously considered, except that it is, perhaps, less closely connected with excitement or exertion, and is often not so amenable to treatment. An abrupt throbbing impulse is more frequently absent than present.

(*d*) Palpitation is sometimes due to mechanical displacements of the heart, as by pleuritic fluid, pericardial effusion, and tumors of the chest and abdomen. The patient feels the beating of his heart; but physical evidence of deranged action is very frequently absent.

(*e*) Palpitation occurs as a prominent sympton in certain conditions of the blood, as in simple anæmia, leucocythæmia, in gout, and especially in exophthalmic goitre. It is also occasioned by the presence in the blood of substances introduced from without, as in the case of tobacco-smoking and the use of strong tea.

(*f*) The cases comprised in this group are characterised by the occurrence of palpitation in definite attacks of sudden access, and by the proportion which is preserved between the perversion of the heart's action and the patient's sensations; and also by the evidence of the disturbed inervation of organs connected with the heart. They form the transition, as it were, from palpitation to angina pectoris, and present some analogy to tic, both of the neuralgic and painless kinds. The attack commences often without obvious cause; the heart's contractions are generally increased in frequency, intensity, and abruptness; they may be regular and violent, or exhibit the extreme of disorder. The pain may vary from slight soreness and uneasiness at the præcordia to extreme agony, associated with the impression of impending death; and a sick and faint feeling with one may, with another, become repeated vomiting and actual syncope. Griping pains in the abdomen and diarrhœa are sometimes present. Such attacks are met with in gout, exophthalmic goitre, rheumatic inflammation of the heart and its membranes, in cyanosis, and sometimes when there is no discoverable disease. That they are neuroses of the heart is rendered probable by their analogy with tic just referred to, and with those forms of neuralgia of sudden access; and this is especially the case when no changes in the heart can be detected. The very frequent, abrupt, regular action of the heart seen in an animal, as the dog, after section of the pneumogastric nerves, recalls such cases to the mind, and prompts the question whether they may not be due to temporary and partial suspension of the functions of these nerves.

The cases briefly related below, two of which I examined after death, illustrate this form of palpitation.

*Palpitation; hypertrophy and dilatation of the heart; pericarditis.*—J. H——, aged twenty-three, was a patient in University College Hospital in the vacation of 1867. He states that two years ago he was confined to the house for a week with pains in his limbs. Eleven days before admission he was at work in his usual health, when he was seized with sudden pain over the heart, with faintness, and with a sensation of palpitation. These symptoms somewhat abated, but nine days afterwards he was observed to be rather livid. On admission there was slight lividity, and the veins of the neck were a little distended. Præcordial pain and dyspnœa were severe, and vomiting, which had occurred previously, was now frequent. The heart's impulse was seen over a wide area, from outside the nipple line to the sternum; and the præcordial region was unduly prominent. To the hand the impulse was violent and irregular in the highest degree, and a loud systolic murmur was heard both at the apex and at the base. During the night the pain and restlessness were extreme. The next day the face appeared somewhat swollen; the head and hands were sweating profusely, whilst the rest of the body was dry and cool. The pain became agonising, but was greatly relieved by hyperdermic injections of morphia, repeated from time to time. Two days after, and fifteen from the commencement of the attack, the patient died. At the post-mortem examination eight ounces of orange-colored fluid were found in the pericardium ; and on the heart, about the root of the great vessels, was a little recent lymph. The heart was greatly dilated and hypertrophied, especially on the left side. The valves were stained yellow. There was considerable thickening of the aortic and mitral valves, but no deposits of fibrin were found on these or on the other valves, which were quite healthy ; nor was there rupture either of the segments or of any of the valvular apparatus. The blood was fluid in the heart, or very recently coagulated ; and no ante-mortem clots were met with either here or in the pulmonary vessels or in the great vessels of the neck. The extreme severity of the symptoms, and the absence of post-mortem evidence of a sufficient cause, point to the nervous origin of the palpitation in this case, which, in truth. differs little from angia.

*Cyanosis, with attacks of palpitation.*—J. D——, aged two years, was a patient in the York Dispensary. The mother said the child had been bluish almost from birth, and had been subject to fits of sickness and pain, in which its color became darker and mottled. The light hair and other peculiarities of cyanotic children were present in a marked degree. Together with evidence of considerable enlargement of the heart, there was a loud, sharp, systolic murmur heard all over the front of the chest, but with greatest intensity at the mid-sternum, opposite to the interval between the third and fourth left cartilages. On three occasions the child was brought to me in a "fit" which presented the appearance of severe cardian dyspnœa and angina ; the whole surface was intensely cyanotic ; the heart was felt beating very frequently, abruptly, and with some violence, but quite regularly ; the murmur, however, had disappeared—an observation which was verified during another attack, six days afterwards—whilst it was loud and distinct the next day, when the heart was acting quietly. Violent purging accompanied these attacks. The child died six weeks afterwards.

At the post-mortem examination, the heart was found considerably dilathed and hypertrophied, especially the right ventricle. At the base of the septum ventriculorum was an opening the size of a hazel-nut, and immediately over it arose a single vessel ·7 of an inch in diameter. The valves of this aorto-pulmonary vessel were somewhat thickened, and the mitral and tricuspid valves were nodulated from old inflammation. No mechanical cause was found to account for the attacks of palpitation and of dyspnœa, nor for the absence of murmur during their occurrence. The foramen ovale was oblique. and just permitted a surgeon's probe to pass through it.

The third case is that of a gentleman aged fifty-nine, who, but for attacks of palpitation, enjoys good health, and is not the subject of gout or other disease. He has had occasional attacks of palpitation for some years, but recently they have assumed especial severity and recur every two, three, or four weeks. They commence without premonitory symptoms, except perhaps a trifling sensation at the præcordia, the patient having felt particularly well for a day or two previously. Suddenly the heart begins to throb with some violence and with remarkable frequency, and continues to do so perhaps for some hours, when, as the sufferer himself informs me, the action becomes irregular and intermittent, and in time, again rapid and abrupt as at the commencement. A feeling of sickness and faintness is present, but vomiting is rare, though it shortens the attack, and has on this account been induced by mustard and water. Syncope has now and then occurred. When the attack has lasted some hours, a feeling of soreness is experienced over the heart, and in time is attended with marked tenderness. Sleep is almost abolished. After thus continuing for three or four days and nights, the palpitation suddenly ceases, its termination being announced by a sensation at the episternal notch. Physical examination of the heart in its period of calm reveals some enlargement of the organ, but murmurs and irregularity of action are entirely absent, except reduplication of the first sound. The pulse has a frequency of 56; its sphygmographic tracing is that of health. During an access of palpitation, the heart's contractions are so rapid as to render the pulse uncountable; no murmur has been present, but the impulse has been rather violent, abrupt, and remarkably frequent, but quite regular, forcibly reminding me of the beating of a dog's heart after division of the pneumogastric nerves.

The association of acute pericarditis and anginal symptoms is peculiar; but a case in some respects similar is related by Andral ("Clin. Med.," vol. iii., p. 19). Attacks of palpitation and dyspnœa occurring in cyanosis are described by Dr. Walshe; but I have not met with a case recorded resembling that last described.

Treatment.—The removal of the immediate *cause*, when this can be ascertained, is the first indication. If strong tea or tobacco-smoking is at fault, this should be prohibited. If acidity of the stomach is the cause, a few grains of bicarbonate of soda, with a little magnesia, will give relief.

*Hygiene and Diet.*—An endeavor should be made to reduce the nervous susceptibility so frequently present, especially in women, by inculcating habits of self-control; and, to this end, change of scene and society, as well as some occupation, are valuable aids. Over-work, of the brain especially, and of the body, should be avoided, and the encouragement of the depressing passions should be firmly discountenanced.

The diet should be adapted to the digestive powers; this is most essential in middle and advanced life, and the irritative dyspepsia often present should be treated. The bicarbonate of soda, with ammonia-citrate of iron, shortly before meals, is very useful for the purpose. Regular and moderate exercise in the open air is desirable.

*Reconstituent remedies* of all kinds are of great service, especially iron, not only in marked cases of anæmia, but whenever the general health and tone of the system are impaired; the solution of the sesquichloride of iron is a convenient form, in doses of from fifteen to thirty minims. In those chronic cases in which the health improves under such treatment, whilst the palpitation persists, I have found the liquor arsenicalis, in doses of three minims and upwards, give relief in two or three days' time. Cod-liver oil may often be given with great advantage.

*The diffusable stimulants and anodynes* are all useful, particularly during attacks of palpitation. They relieve the faint, sick feeling, and the pain; and sometimes lessen the irregular action of the heart, and promote sleep. The most valuable of them for the latter purpose is chloral, in doses of ten to thirty grains, once or twice during the night; since sleep follows its use in a few minutes, and nausea and constipation are not experienced the next day, and very rarely headache. Its continued use does not appear to be attended with inconvenience. Opium is useful, but it is not so well borne; it is best associated with ammonia and spirit of chloroform. Digitalis, except in the obviously weakened and dilated heart, has no marked effect; but, in such cases the infusion, in doses of two drachms and upwards, is most valuable. Of all remedies, I have found the subcutaneous injection of morphia to be the most trustworthy; it nearly always alleviates, and very frequently cures, the complaint after a few repetitions. One-twelfth to one-tenth of a grain is a sufficient dose to begin with, and there is little occasion to exceed this quantity. The injection should be repeated every third or fourth day. Though the arm is the most convenient place to insert the needle, the injection is sometimes more effectual at the præcordia. When morphia fails, or is not tolerated, atropine may be substituted; but is not, I think, so useful. If the prick is objected to, the morphia may be applied to the derma denuded by a small blister raised with ammonia, as Trousseau recommends. The hypodermic application of morphia has the additional advantage of relieving pain.

*Local applications* are of great service. Belladonna and its preparations stand in the first rank of these. The ordinary belladonna plaster, of good size and renewed every week or ten days, is very convenient and effective. It presents three special advantages: it protects the chest walls, which are tender, from the pressure of the clothes, and seems to deaden the painful impulse of the heart; besides this, it impedes slightly the free movement of the parts, and thus obviates the muscular pain often present. Another good application is a mixture of equal parts of belladonna and of liniment of chloroform, sprinkled on cotton wool.

Hot linseed and mustard poultices are soothing, and are easily obtained.

Two methods of direct mediation of the pneumogastric and sympathetic nerves have been proposed, which are steps in the direction of scientific therapeutics, and well deserve study. Dr. Althaus has advocated the application of the galvanic current to these nerves in their course ("Medical Electricity"), and Dr. Augustus Waller their compression in the neck by the thumbs (*Practitioner*).

*Harley street, W., January,* 1871.

---

*Dangerous and Fatal Results from the use of Hydrate of Chloral.*

By H. W. Fuller, M. D., F. R. C. P., Senior Physician to the St. George's Hospital, etc.

---

As the hydrate of chloral is being largely administered as a sedative and hypnotic, and is often recklessly employed in excessive doses, as if it possessed no noxious properties, it may be well to record a few cases in which moderate doses were productive of dangerous and even fatal results.

On February 9th, 1870, J. S.—— was admitted under my care into St. George's Hospital, suffering from slight anasarca and bronchitis connected with chronic Bright's disease. He was restless and nervous and unable to sleep, and therefore, after he had been some days in the hospital, and was exhausted for want of sleep, I ordered the chloral draught of the hospital (containing thirty grains of chloral) to be taken at bedtime. Soon after he had taken it he jumped up in bed, clutched at his heart, and complained that the medicine produced a sense of burning. In the course of a few minutes he became violently delirious, and though after a time the delirium subsided, so much depression ensued that Dr. Jones, our resident officer, had great difficulty in sustaining his heart's action. Gradually, however, the heart recovered itself, the pulse returned at the wrist, and in a few hours he was out of danger.

Having just read M. Liebreich's assertion that hydrate of chloral, when in contact with an alkali, is transformed into chloroform and formic acid, it occurred to me that the extraordinary results witnessed in my patient's case might be attributable to an alkaline condition of the stomach, whereby the chloral was at once converted into chloroform, and thus induced the symptoms which were observed. I therefore determined to try it once again, taking care, on this occasion, to guard against such an occurrence by administering the chloral in combination with a full dose of acid. The result, however, was precisely the same as on the first

